# Agathisflavone Modulates the Kynurenine Pathway and Glial Inflammatory Responses with Implications for Neuroprotection

**DOI:** 10.3390/ijms262411951

**Published:** 2025-12-11

**Authors:** Deivison Silva Argolo, Lucas Matheus Gonçalves Oliveira, Cleonice Creusa dos Santos, Lilian Vanessa da Penha Gonçalves, Erick Correia Loiola, Bruno Solano de Freitas Souza, George E. Barreto, Arthur Morgan Butt, Jorge Mauricio David, Alexsandro Branco, Isabella Mary Alves Reis, Annabel Azevedo-Silva, Silvia Lima Costa, Maria de Fátima Dias Costa

**Affiliations:** 1 Laboratory of Neurochemistry and Cellular Biology, Department of Biochemistry and Biophysics, Institute of Health Sciences, Federal University of Bahia, Av. Reitor Miguel Calmon S/N, Salvador 40231-300, BA, Brazil; dsaargolo@gmail.com (D.S.A.); lucas.nom@gmail.com (L.M.G.O.); cleonicemev@gmail.com (C.C.d.S.); lilianvpnha@gmail.com (L.V.d.P.G.); 2 Center of Biotechnology and Cell Therapy, São Rafael Hospital, D’Or Institute for Research and Education (IDOR), Av. São Rafael, 2152-São Rafael, Salvador 41253-190, BA, Brazil; erickloi@gmail.com (E.C.L.); bruno.solano@idor.org (B.S.d.F.S.); 3 Neuroendocrine Pharmacology Lab, Department of Biological Sciences, Faculty of Science and Engineering, University of Limerick, V94 T9PX Limerick, Ireland; george.barreto@ul.ie; 4 School of Medicine, Pharmacy and Biomedical Sciences, University of Portsmouth, Portsmouth PO1 2UP, UK; arthur.butt@port.ac.uk; 5 Department of General and Inorganic Chemistry, Institute of Chemistry, University Federal da Bahia, Salvador 40170-110, BA, Brazil; jmdavid@ufba.br; 6 Laboratory of Phytochemistry, Department of Health, State University of Feira de Santana, Av. Transnordestina, s/n, Feira de Santana 44036-900, BA, Brazil; branco@uefs.br (A.B.); imareis@uefs.br (I.M.A.R.); annabelfarma@gmail.com (A.A.-S.); 7 National Institute of Translational Neuroscience (INCT-CNPq), Federal University of Rio de Janeiro, Rio de Janeiro 21941-90, RJ, Brazil

**Keywords:** tryptophan, kynurenines, agathisflavone, IDO1, glial cell

## Abstract

The cells in the central nervous system (CNS) can adapt to injury and inflammation through structural and functional changes, many of which are mediated by the kynurenine pathway (KP). Studies using glia–neuron co-cultures showed that the biflavonoid agathisflavone (FAB), purified from the leaves of *Cenostigma pyramidale* Tul., a plant native to the Brazilian caatinga, exerts strong neuroprotective effects. This study evaluated whether agathisflavone (1 µM) modulates these responses in human and murine astrocytes and microglia exposed to inflammatory activation with lipopolysaccharide (LPS, 1 µg/mL), excitotoxic activation of NMDA receptors with quinolinic acid (QUIN, 500 µM), or inhibition of the KP rate-limiting enzyme indoleamine 2,3-dioxygenase 1 (IDO1) with 1-methyl tryptophan (1-MT, 1.5 μM). Co-treatment with FAB increased astrocyte viability relative to LPS, QUIN, or 1-MT alone, by up to 35% (*p* < 0.05), while reducing GFAP overexpression and other features of reactive astrogliosis. FAB decreased the proportion of Iba-1^+^ microglia, indicating anti-inflammatory effects. When combined with QUIN or 1-MT, FAB reversed the elevation of iNOS (*p* < 0.0001) and reduced IL1β upregulation. FAB also modulated KP activity in a cell type-specific manner. In astrocytes, FAB with QUIN or with 1-MT increased IDO activity, whereas in microglia, FAB alone reduced it. In microglia, kynurenine-3-monooxygenase (KMO) expression was significantly increased under FAB+QUIN or FAB+1-MT (*p* < 0.0001). Finally, astrocyte-conditioned medium from FAB-treated cells increased the viability of neuron-like PC12 cells by up to 40%. Collectively, these findings show that FAB confers cytoprotective and anti-inflammatory actions on glial cells, modulates KP signalling in a context-dependent manner, and supports neuronal survival under neuroinflammatory conditions.

## 1. Introduction

In the CNS, astrocytes and microglia play essential immunological roles and communicate through complex signalling networks, maintaining homeostasis and shaping the inflammatory responses. One pathway of particular importance is the kynurenine pathway (KP), which is the main route for the catabolism of the essential amino acid tryptophan (Trp). The KP generates several neuroactive metabolites that can either be neuroprotective or neurotoxic, and thus it directly influences the outcomes of neuroinflammation [1,2,3,4,5].

Indoleamine 2,3-dioxygenase 1 (IDO1), the first and rate-limiting enzyme of the KP, is activated by pro-inflammatory cytokines such as tumour necrosis factor (TNF), interleukin-1β (IL-1β), and interferons (IFNs), as well as by bacterial lipopolysaccharide (LPS). IDO1 converts Trp into L-kynurenine (KYN), which is further metabolized into downstream compounds, including quinolinic acid (QUIN) [6,7,8,9,10,11]. QUIN is a potent agonist of N-methyl-D-aspartate (NMDA) receptors, and excessive QUIN production has been closely linked to neuronal excitotoxicity and neuroinflammation. QUIN biosynthesis depends largely on kynurenine monooxygenase (KMO), an enzyme expressed in activated microglia [12]. Indeed, microglia are a main cell type producing QUIN in the CNS. By contrast, astrocytes produced a large amount of KYN that is then catabolised into kynurenic acid (KYNA), an NMDA receptor antagonist, by the kynurenine aminotransferase (KAT) enzymes [13]. Activation of the KAT enzymes in astrocytes is considered a neuroprotective mechanism as KYNA can limit glutamatergic excitotoxicity, a feature common to traumatic brain injury, ischemia, and neurodegenerative diseases [6,7,8,9,10,11,12,13,14].

Studies using glia–neuron co-cultures showed that agathisflavone (bis-apigenin or FAB)—a biflavonoid isolated from *C. pyramidale* Tul. (synonyms *Poincianella pyramidalis*, *Caesalpinia pyramidalis*), a plant native to the Brazilian caatinga—exerts strong neuroprotective effects [15]. In vitro, FAB reduces glutamate-induced excitotoxicity and shifts activated microglia toward a less inflammatory phenotype [15,16]. FAB limits microglial proliferation and decreases production of pro-inflammatory molecules such as TNF and IL-1β, while preserving neuronal integrity, promoting neurite outgrowth, and increasing the anti-inflammatory cytokine IL-10 [15]. FAB also supports axonal myelination by stimulating oligodendrocytes, likely through its modulation of microglial inflammatory signalling [17], supported by evidence that FAB dampens microglial activation by inhibiting the NLRP3 inflammasome, reducing IL-1β expression, and modulating microRNAs (miRNAs) via STAT3-dependent pathways [18,19]. Together, these findings suggest that FAB could represent a promising adjuvant for treating neurodegenerative diseases [18,19]. Here, we provide evidence that FAB acts via the KP to modulate glial responses to inflammatory and excitotoxic stimuli and identify potential biomarkers and therapeutic targets within this pathway.

## 2. Results

### 2.1. Modulation of Astrocyte and Microglial Toxicity and Reactivity by Agathisflavone and Kynurenine Pathway Agents

FAB primarily preserves glial viability and regulates how these cells respond to inflammatory and excitotoxic stress. We first measured cell viability using the MTT assay, a colorimetric assay measuring mitochondrial dehydrogenase activity. Cells were exposed to FAB (1 µM) alone or together with *Escherichia coli* lipopolysaccharide (LPS, 1 µg/mL) for 24 h. To test whether FAB modulates the kynurenine pathway (KP), we also treated cultures for 24 h with FAB (1 µM) in the presence of QUIN (500 µM), an N-methyl-D-aspartate (NMDA) receptor agonist that induces excitotoxicity, or 1-methyl-DL-tryptophan (1-MT, 1.5 μM), a competitive inhibitor of IDO1.

**Figure 1:**
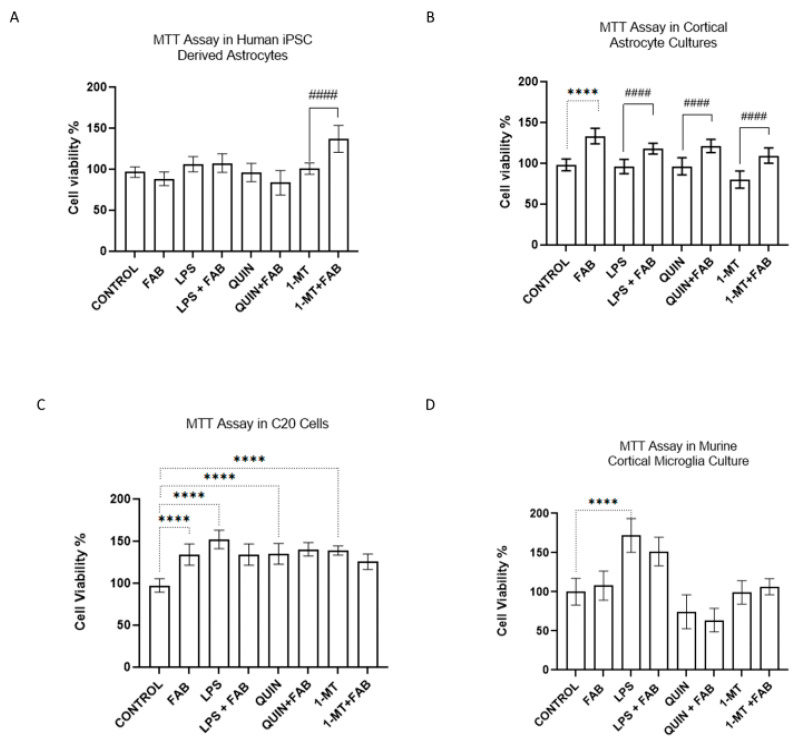
Effects of agathisflavone (FAB) on the viability of glial cells under inflammatory conditions. (**A**) Human iPSC-derived astrocytes (hAst), (**B**) primary murine cortical astrocytes, (**C**) human C20 microglial cell line, and (**D**) primary murine microglia were treated for 24 h with lipopolysaccharide (LPS, 1 µg/mL), quinolinic acid (QUIN, 500 µM), 1-methyl-D-tryptophan (1-MT, 1.5 μM), alone or in combination with FAB (1 µM). Cell viability was assessed by the MTT assay and expressed as % relative to the untreated control, considered 100%. Data represent mean ± SEM (*n* = 3 independent experiments). Statistical analysis: one-way ANOVA followed by Student–Newman–Keuls test. **** *p* < 0.0001 vs. control; #### *p* < 0.0001 vs. respective stimulus without FAB.

In human astrocyte (hAst) cultures, FAB, LPS, QUIN, or 1-MT alone, and FAB combined with LPS or QUIN, did not alter the cell viability (Figure 1A). Interestingly, co-treatment of FAB with 1-MT resulted in a significant (*p* < 0.05) increase in mitochondrial dehydrogenase activity (Figure 1A). In primary astrocytes, co-treatment with FAB and LPS, QUIN, or 1-MT consistently increased viability compared with each stimulus alone (Figure 1B). In C20 microglia, FAB, LPS, QUIN, or 1-MT, alone or in combination, significantly increased MTT reduction (*p* < 0.0001). In primary microglia, an increase in MTT-metabolizing cells was observed after LPS and following FAB treatment (Figure 1C,D). Together, these findings point to the cytoprotective effect of FAB, potentially linked to KP modulation in astrocytes.

Astrogliosis is a hallmark of the CNS inflammatory response, marked by phenotypic, morphological, and molecular changes that support tissue protection and repair [20]. One of the main biomarkers of this process is glial fibrillary acidic protein (GFAP), an intermediate filament of the astrocytic cytoskeleton, constitutively expressed and widely used as an indicator of astrocyte activation [20,21]. We therefore examined whether FAB modulates inflammation- or toxicity-induced astrogliosis and the involvement of the KP, assessing astrocyte morphology and GFAP expression by immunofluorescence in hAst and in primary astrocyte cultures.

**Figure 2:**
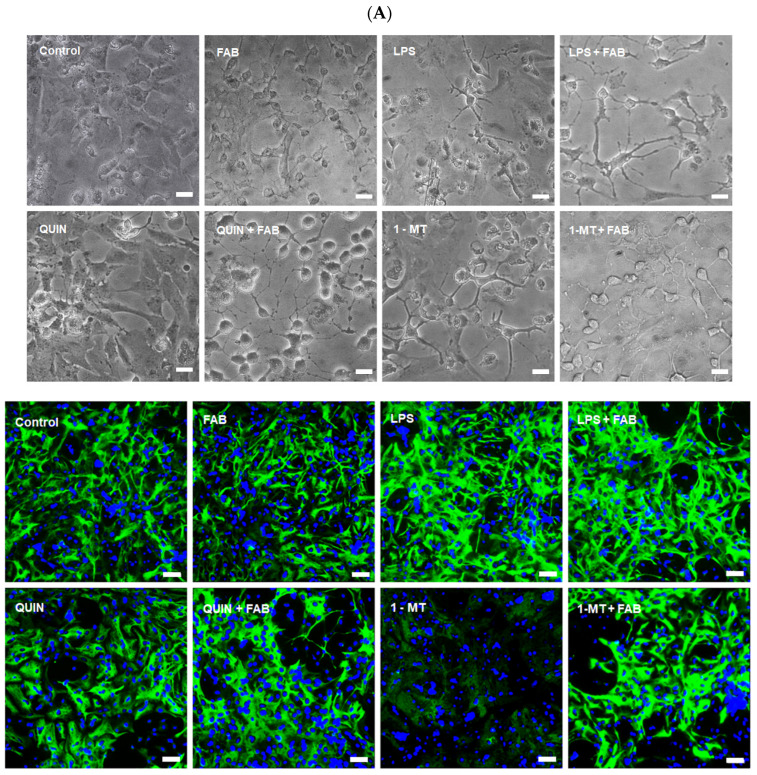

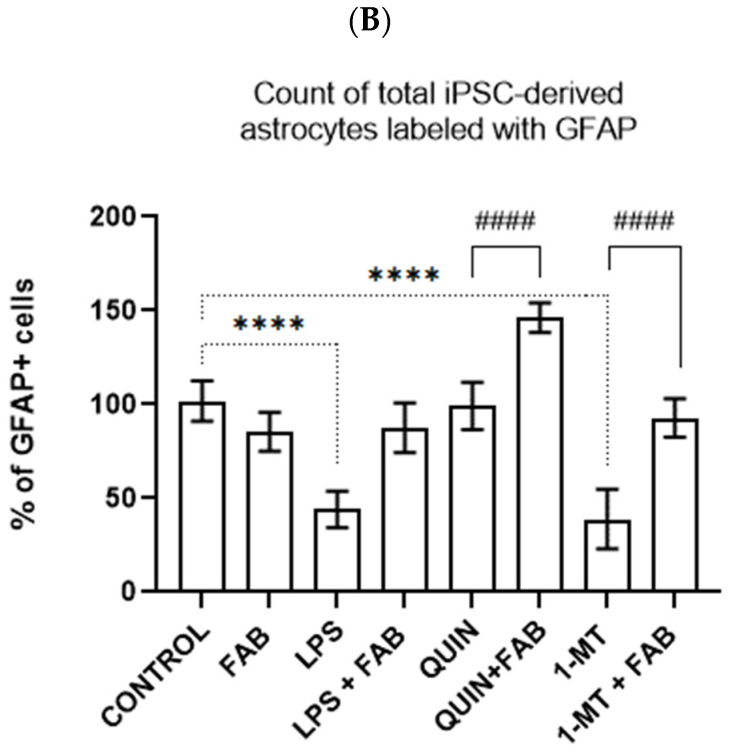
GFAP expression and morphology of human astrocytes treated with FAB and inflammatory stimuli. (**A**) Representative images of astrocytes under phase contrast microscopy (upper panels) and following GFAP immunostaining (green, lower panels) after treatments with LPS, QUIN, 1-MT, or combinations with FAB. (**B**) Quantification of GFAP^+^ cells as % relative to untreated control. Data are mean ± SD (*n* = 3 independent experiments). Scale bar: 50 μm. Statistical analysis: one-way ANOVA followed by Bonferroni’s test; **** *p ≤* 0.0001 vs. control; #### *p* < 0.0001 vs. respective stimulus without FAB.

In hAst, FAB alone did not cause visible morphological alterations; astrocytes remained polygonal and flat, with GFAP evenly distributed in the cytoplasm and short cellular processes, closely resembling untreated controls (Figure 2A,B). In contrast, LPS (1 µg/mL) induced a reactive phenotype with cytoplasmic retraction, elongated processes, and reduced GFAP expression. Similar changes followed 1-MT or QUIN treatment, which did not affect GFAP expression levels. In hAst, exposure to FAB in association with LPS, QUIN, or 1-MT did not change GFAP expression levels, although morphological changes were observed, suggesting that FAB may exert a modulatory effect on inflammation-driven astrogliosis and help prevent its progression (Figure 2A,B).

**Figure 3:**
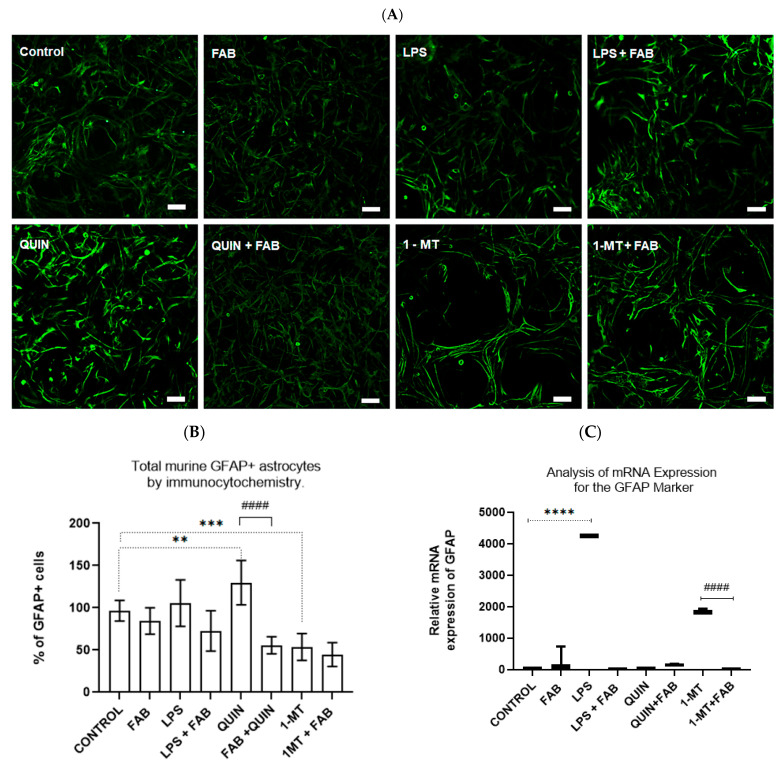
GFAP expression and morphology in murine primary cortical astrocytes treated with FAB and inflammatory stimuli. (**A**) Representative immunofluorescence images of GFAP (green), quantitative GFAP+ (**B**,**C**), and mRNA expression of GFAP assessed by RT-qPCR. Data are mean ± SD (*n* = 3 independent experiments). Scale bar: 50 μm. Statistical analysis: one-way ANOVA followed by Bonferroni’s test; ** *p* ≤ 0.005 vs. control, *** *p* ≤ 0.0005 vs. control, **** *p* ≤ 0.0001 vs. control; #### *p* < 0.0001 vs. respective stimulus without FAB.

In primary astrocytes, control and FAB-only (1 μM) conditions showed the expected polygonal morphology with basal GFAP localized in the soma and short processes. LPS (1 μg/mL) provoked reactive changes consistent with astrogliosis, cell body shrinkage, elongated processes, and increased GFAP immunoreactivity (Figure 3A,B). QUIN (500 μM) and the IDO inhibitor 1-MT (1.5 μM) produced similar alterations (Figure 3A,B). Both LPS and QUIN significantly increased GFAP levels (*p* ≤ 0.05), and these effects were attenuated by co-treatment with FAB. RT-qPCR confirmed GFAP transcripts after LPS or 1-MT, which was not observed when FAB was co-applied (Figure 3C).

**Figure 4:**
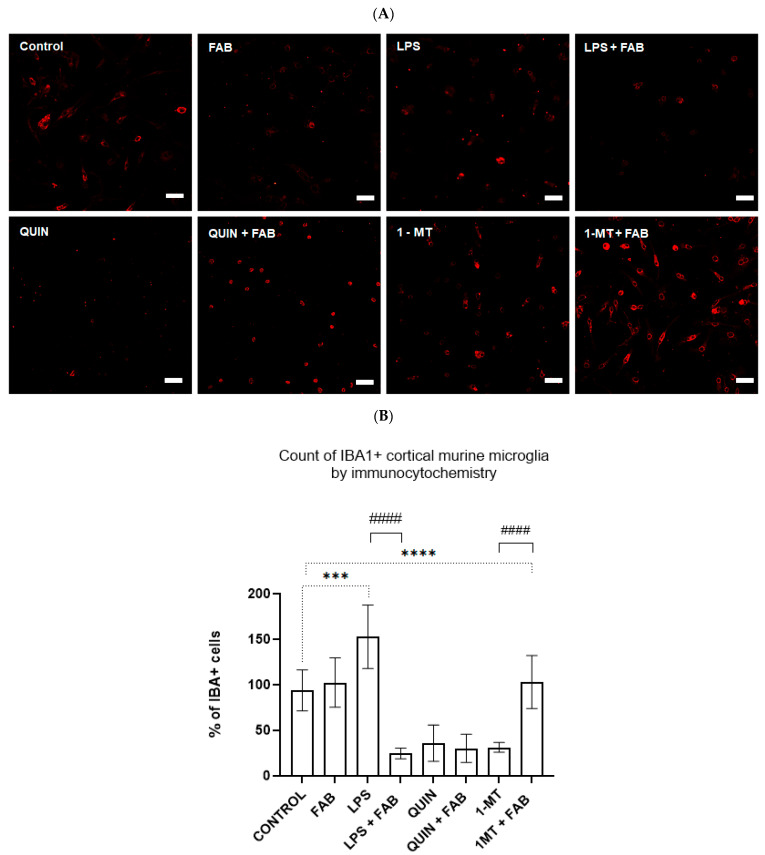
Morphology and proliferation of murine microglia treated with FAB and subjected to inflammatory and KP-related stimuli. (**A**) Representative images of microglia immunofluorescence labelled with the microglial marker IBA-1 (red). (**B**) Quantification of IBA-1^+^ cells expressed as % relative to control. Data are mean ± SD (*n* = 3 independent experiments). Scale bar: 50 μm. Statistical analysis: one-way ANOVA followed by Bonferroni’s test; *** *p ≤* 0.0005 vs. control, **** *p ≤* 0.0001 vs. control. #### *p* < 0.0001 vs. respective stimulus without FAB.

To characterize microglial activation, mouse microglia were treated with FAB (1 µM), LPS, QUIN, and 1-MT, and immunolabeled for Iba-1, a calcium-binding adaptor protein expressed by microglia/macrophages, in association with the Ki67 marker of proliferation (Figure 4A,B). LPS significantly increased (*p* < 0.0001) the proportion of ameboid IBA-1^+^ cells, consistent with a reactive, proliferative phenotype. In contrast, FAB reduced proliferating microglia, indicating an anti-inflammatory or deactivating effect on microglia. QUIN or 1-MT also reduced the proportion of Iba-1^+^ cells (*p* < 0.001%), and a similar reduction was seen with FAB+QUIN, suggesting that QUIN broadly affects microglial viability/activation in ways not fully prevented by FAB. Conversely, inhibition of IDO by 1-MT treatment was associated with an increase in Iba-1^+^/Ki67+ cells and clear morphological signs of activation, supporting a key role for IDO in regulating neuroinflammatory responses in astrocytes and microglia.

### 2.2. Agathisflavone Modulates the Gene Expression of Inflammatory Markers Through KP

**Figure 5:**
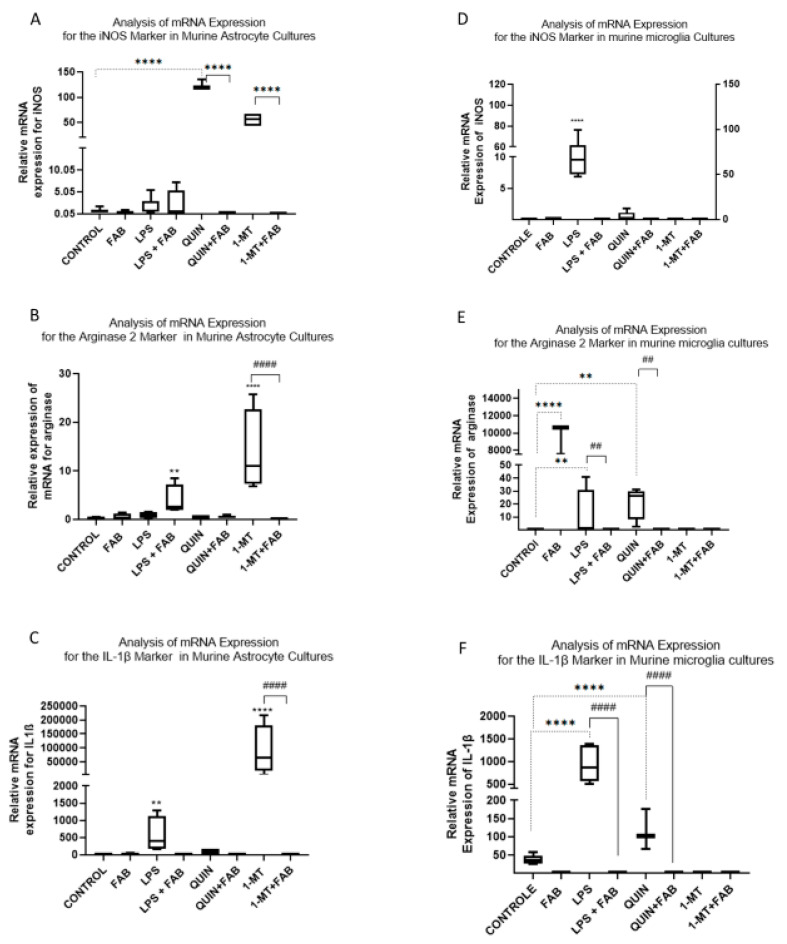
Gene expression of inflammatory agents in cultures of astrocytes (**A**–**C**) and primary cortical microglia (**D**–**F**). The relative mRNA levels of iNOS, ARG2, and IL-1β were determined by RT-qPCR after 24 h of treatment with LPS, QUIN, l-MT, FAB, or their combinations. Results are expressed as fold change from control (mean ± SD, *n* = 3 independent experiments). Statistical analysis: one-way ANOVA followed by Bonferroni’s test. ** *p* < 0.005 **** *p* < 0.0001 vs. control; ## *p* < 0.005 #### *p* < 0.0001 vs. the respective stimulus without FAB.

Regulation of gene expression is central to how cells adapt to environmental and inflammatory cues. We next tested whether FAB regulates the expression of inflammatory and metabolic genes in glia and whether it shapes the KP. In primary mouse cortical astrocytes, iNOS expression differed robustly across conditions (*p* < 0.0001). FAB alone did not change iNOS, whereas LPS, QUIN, or l-MT each increased iNOS vs. control (Figure 5A). This increase was also present in combined LPS+FAB treatment but was not observed when FAB was combined with QUIN or with l-MT (Figure 5A). For ARG2, FAB+LPS significantly increased expression relative to LPS alone and to the control. 1-MT also elevated ARG2 expression (*p* < 0.0001; Figure 5B), and this effect was not observed by co-treatment with FAB. Notably, FAB+LPS produced higher ARG2 expression than LPS alone. For IL1β, the highest-level expression occurred with LPS and with 1-MT (*p* < 0.0001). Adding FAB to either treatment significantly reduced IL1β expression toward control. QUIN also elevated IL1β, whereas FAB+QUIN brought expression back to control-like levels (Figure 5C). In primary cortical microglia, LPS strongly induced iNOS, ARG2, and IL1β expression (*p* < 0.0001), and FAB markedly attenuated these increases (Figure 5D–F). Although LPS alone did not significantly change ARG2 expression, LPS+FAB did increase it vs. control (*p* < 0.05). LPS and 1-MT elevated IL1β expression (*p* < 0.001 and *p* < 0.0001, respectively), while co-treatment with FAB restored expression levels close to controls. Collectively, these data indicate that LPS and QUIN are potent inducers of inflammatory gene expression in astrocytes and microglia, and that FAB counteracts this activation, with IDO inhibition exhibiting a distinct regulatory profile.

**Figure 6:**
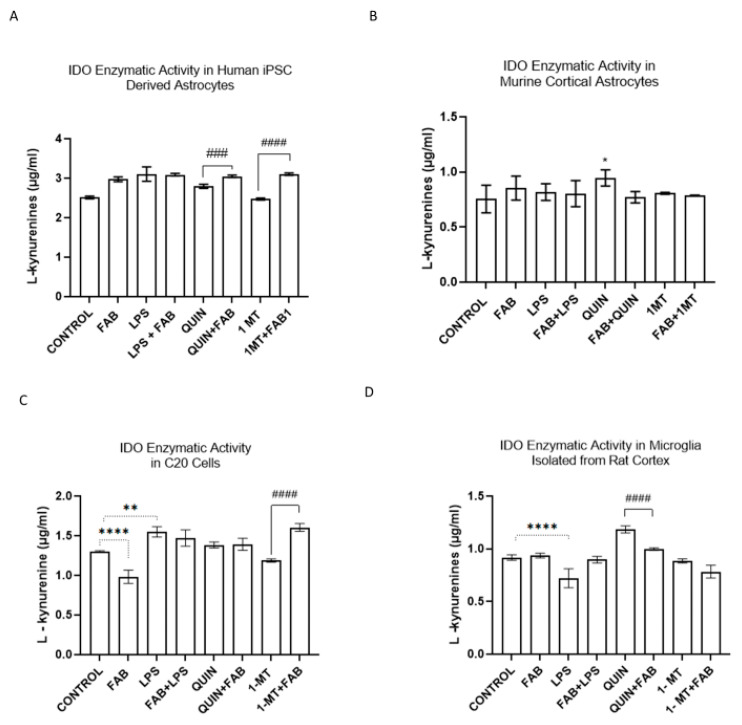
Evaluation of IDO activity in glial cells treated with FAB and KP modulators, measured by L-kynurenine levels in cultures of (**A**) hAst, (**B**) primary murine astrocytes, (**C**) C20 microglia, and (**D**) primary microglia, after 24 h treatment with LPS, QUIN, 1-MT, FAB, or combinations. Statistical analysis: one-way ANOVA followed by Bonferroni’s test. Data are presented as mean ± SEM (*n* = 3 independent experiments); * *p* < 0.05 vs. control; ** *p* < 0.005 vs. control; **** *p* < 0.0001 vs. control; #### *p* < 0.0001 vs. the respective stimulus without FAB.

To test KP activity, we quantified L-kynurenine levels in astrocytes and in microglia culture medium as an indicator of the IDO enzymatic activity. In both hAst and primary murine cortical astrocytes, FAB, LPS, or QUIN alone significantly increased L-kynurenine levels (Figure 6A,B). In hAst, FAB+QUIN or FAB+1-MT further enhanced L-kynurenine levels (*p* < 0.0001), suggesting a potentiating effect under these conditions on IDO activity (Figure 6A). In the C20 human microglial cells, FAB significantly reduced L-kynurenine levels, whereas they were increased by LPS and FAB+1-MT (*p* < 0.0001). QUIN also increased L-kynurenine levels, and this elevation persisted in the presence of FAB (Figure 6C). In primary microglia, QUIN significantly increased L-kynurenine levels, but the increase was attenuated when QUIN was combined with FAB (Figure 6D), indicating FAB can modulate IDO during inflammatory or excitotoxic conditions.

To understand how FAB and KP modulators redirect Trp metabolism, we measured transcripts for IDO, KMO, and KAT (Figure 6A–D). In primary astrocytes, QUIN significantly increased IDO expression (*p* < 0.0001) versus control (Figure 6A). FAB, LPS, LPS+FAB, QUIN+FAB, 1-MT, or 1-MT+FAB did not produce additional significant changes in IDO expression, suggesting that these stimuli, whether applied alone or in combination, are insufficient to induce significant transcription of this gene (Figure 6A). KAT mRNA was not altered by FAB (alone or in combination treatments), whereas 1-MT increased KAT, consistent with a compensatory shift toward the protective KYN → KYNA branch when IDO is inhibited (Figure 6B). In microglia, FAB and QUIN were the most effective inducers of IDO (Figure 6C), whilst KMO was significantly upregulated by LPS but not changed by QUIN or 1-MT alone, and KMO was also significantly upregulated when FAB was combined with QUIN or with 1-MT (*p* < 0.0001) (Figure 6D).

### 2.3. Neuroprotective Effect of the Secretome from Astrocytes Treated with Agathisflavone and Kynurenine Pathway Modulators on Differentiated PC12 Cells

**Figure 7:**
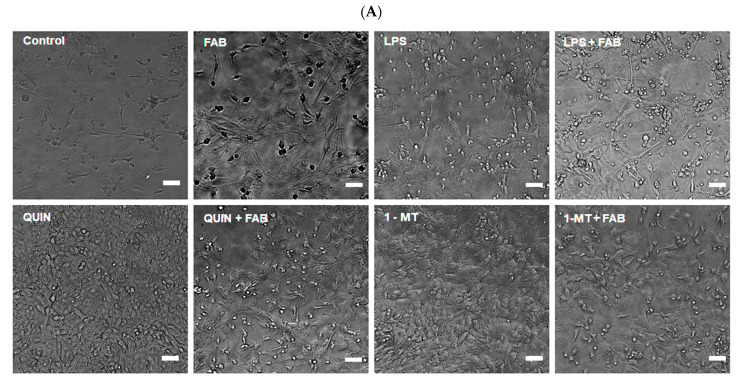

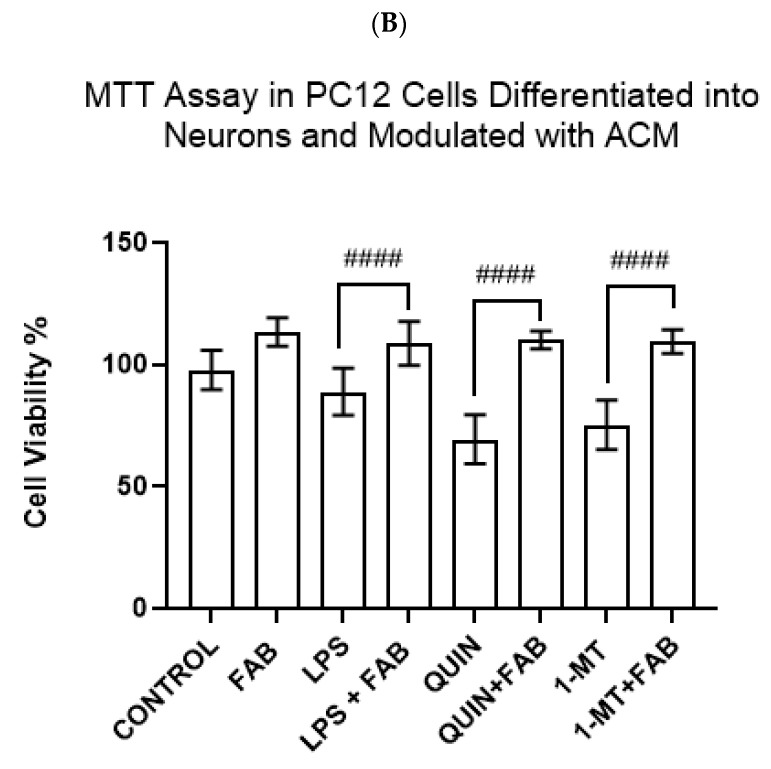
Neuroprotective effects of astrocyte-derived conditioned medium (ACM) on differentiated PC12 cells. (**A**) Representative phase-contrast images showing PC12 cell morphology. (**B**) MTT assay showing viability of PC12 cells after exposure to ACM from astrocytes treated with LPS, QUIN, 1-MT, FAB, or combinations. Data represent mean ± SD (*n* = 3 independent experiments). Scale bar: 50 μm. Statistical analysis: one-way ANOVA followed by Bonferroni’s test; #### *p* < 0.0001 vs. the respective stimulus without FAB.

The results above indicate that FAB modulates glial responses to LPS, largely through the KP. To test whether this glial modulation translates into neuroprotection, we exposed neuron-like PC12 cells to astrocyte-conditioned medium (ACM) generated under the different conditions. ACM was collected from murine astrocytes treated with vehicle (DMSO, control), LPS (1 µg/mL), FAB (1 µM), QUIN (500 µM), 1-MT (1.5 μM), or the indicated combinations. After 24 h, we assessed PC12 viability (MTT assay) and morphology (phase-contrast microscopy) (Figure 7A). PC12 cells exposed to ACM from LPS-treated astrocytes displayed morphological signs of cellular damage, including soma swelling. By contrast, ACM from astrocytes treated with QUIN, either alone or together with FAB, preserved features typical of undifferentiated PC12 cells, with minimal neurite extension, suggesting these media did not support or maintain neuronal differentiation. Interestingly, differentiated PC12 cells exposed to ACM from astrocytes treated with FAB + the IDO inhibitor 1-MT maintained a neuronal phenotype, with polarized shapes, well-defined cell bodies, and an extensive neurite network. In viability assays, ACM from LPS, QUIN, or 1-MT alone significantly reduced the survival of differentiated PC12 cells (Figure 7B). In contrast, ACM from FAB-treated astrocytes, whether FAB was given alone or combined with LPS, QUIN or 1-MT, increased PC12 viability (*p* < 0.0001).

### 2.4. Detection of Tryptophan Metabolites in Media from Astrocytes, Microglia and PC12 Cells

To further evaluate how FAB influences Trp metabolism and KP activity under our experimental stimuli conditions, we used high-performance liquid chromatography (HPLC) to detect, and where possible, quantify key KP metabolites. Reliable detection of these metabolites is essential for interpreting biochemical shifts linked to glial modulation and inflammation. HPLC analysis revealed multiple compounds consistent with Trp-derived metabolites in all cellular samples analyzed. Identification relied on analytical standards that produced well-resolved peaks at characteristic retention times (RTs): Trp (~1.1–1.3 min), KYN (~8.1–8.2 min), QUIN (~10.6–10.7 min), and 1-methyl-D-tryptophan (1-MT, ~14.5–14.7 min). Using these standards, we profiled chromatograms from ACM, microglia-conditioned medium (MCM), and supernatants from differentiated PC12 cells exposed to ACM or MCM across the various conditions. RTs were consistent across samples, supporting the presence of structurally identical (or closely related) compounds. The Trp peak was abundant and sharply resolved in most samples, indicating high Trp availability. Robust peaks for KYN (~8.1 min) and QUIN (~10.6 min) further supported active KP flux. We also detected longer-RT peaks (~14.5–23.0 min) compatible with less-polar KP derivatives, potentially including KYNA. These identifications were supported by the overlap of UV-Vis absorption spectra between sample peaks and the reference standards. Chromatographic profiles were reproducible across independent biological replicates, underscoring the robustness and reliability of the analytical method employed. These findings indicate that our experimental conditions preserve KP activity and validate the model for studying Trp metabolism in glia.

Comparative analysis of ACM, MCM, and PC12 supernatants confirmed active Trp catabolism in both astrocytes and microglia. Although each glial type is often associated with preferential production of either QUIN or KYNA, both metabolites were detectable in the media of astrocytes and microglia. Importantly, samples from LPS, FAB, or FAB+LPS conditions displayed additional chromatographic peaks that did not match our standards. These peaks were absent in controls and in groups treated with the IDO inhibitor 1-MT, suggesting they may represent intermediate or alternative KP metabolites, such as anthranilic acid or 3-hydroxykynurenine. Overall, these chromatographic data confirm that the Trp → KP metabolic pathway is active under all tested conditions and reveal shifts in KP metabolism in response to FAB and to inflammatory or excitotoxic challenges (Supplementary Figure S1).

## 3. Discussion

Our data show that FAB meaningfully reshapes glial responses to inflammatory challenges. In both astrocytes and microglia, FAB reduced pro-inflammatory gene expression, attenuated morphological changes associated with reactive activation, and preserved viability in the presence of LPS or QUIN. These findings align with the view that glial activation is dynamic, where astrocytes and microglia can initially protect after injury but, if overactivated, contribute to neurotoxicity and neuronal loss [22,23,24]. The ability of FAB to prevent this pathological progression indicates immunomodulatory and neuroprotective potential [25].

The results identify KP as a central mechanism for the actions of FAB on glial activation. LPS, QUIN, and IDO inhibition altered IDO activity, shifting Trp metabolism toward either neurotoxic or neuroprotective outputs. FAB, in turn, modulated IDO activity and influenced the generation of KYN, KYNA, and QUIN in a cell-type-dependent manner. Consistent with established biology, astrocytes (via KATs) favour KYNA, a metabolite with established neuroprotective properties, whereas microglia (via KMO) are a predominant source of the neurotoxic QUIN [26,27,28]. The balance between these enzymatic branches determines the cellular outcomes [29,30]. In our results, FAB tended to preserve the KYNA-linked branch under inflammatory conditions and limit QUIN-related toxicity, indicating a shift toward a more protective KP mechanism. Effects on KMO appeared to be context-dependent, whereas KAT expression/activity was maintained even during inflammatory stimulation.

Importantly, FAB’s impact extended to glia-to-neuron communication. Conditioned medium from FAB-treated astrocytes or microglia conveyed protection to differentiated PC12 cells, a widely used model for neurotoxicity studies. Neuronal morphology was maintained, and viability improved, including under QUIN exposure. This supports the idea previously reported that FAB not only acts within glia but also reshapes the paracrine milieu of cytokines, trophic factors, and KP metabolites that govern neuronal health [30,31,32]. By restoring this axis toward a balanced, protective microenvironment, FAB may help maintain neural homeostasis [30,31,32,33,34,35,36,37,38,39].

Combining FAB with the IDO inhibitor 1-MT yielded synergistic benefits across models, with higher cell viability and stronger downregulation of inflammatory markers. These results suggest that FAB’s actions intersect with IDO-dependent signalling, potentially modifying IDO activity and/or downstream metabolite signalling rather than only affecting transcription thereby implying a neuroprotective effect [40,41,42,43].

FAB significantly reduced iNOS, ARG2, and IL-1β induced by the stimuli alone in human astrocytes, indicating coordinated modulation of inflammatory and metabolic pathways. Considering that there are species-specific differences in immune signalling as well as in the enzymatic kinetics of the kynurenine pathway, this study included, in addition to murine cells derived from the brain cortex, iPS-derived astrocytes and mutated microglia of human origin. Thus, a more accurate representation of cellular metabolism, inflammatory signalling, and neuroprotective mechanisms could strengthen the translation of the results and their clinical applicability, which has been recommended in preclinical models. Moreover, considering that KP dysregulation contributes to Alzheimer’s disease and amyotrophic lateral sclerosis, where excessive QUIN, reduced KYNA, and redox imbalance are common [7,27,40], FAB’s capacity to restore KYNA production and blunt QUIN-driven excitotoxicity positions it as a promising candidate for chronic inflammatory and neurodegenerative conditions.

Finally, our chromatographic analyses confirmed major Trp catabolites and revealed additional peaks in FAB-treated conditions that were absent in controls, suggesting a rerouting of metabolic flux toward less toxic profiles [5,25]. The detection of putative intermediates, potentially lesser-studied kynurenine derivatives, highlights FAB’s value not only as a therapeutic modulator but also as a probe to uncover molecular targets and biomarkers of KP regulation in neuroinflammation and neurodegeneration, and quantification of the Trp metabolites will be considered in future studies.

## 4. Materials & Methods

### 4.1. Cell Cultures

#### 4.1.1. Primary Glial and Isolated Microglial Cell Cultures

Primary glial cultures were prepared from the cerebral cortex of neonatal Wistar rats (P0–P8), obtained from the animal facility of the Institute of Health Sciences at the Federal University of Bahia (Salvador, BA, Brazil), following approval from the local Animal Ethics Committee (Protocol CEUA-ICS #6731220818). Briefly, after decapitation, the cerebral hemispheres were dissected, and the meninges were removed. The tissue was mechanically dissociated using a Pasteur pipette and seeded into poly-D-lysine-coated culture flasks in DMEM medium (Gibco, Grand Island, NY, USA) supplemented with 10% fetal bovine serum (FBS), 10% equine serum (EHS), and antibiotics (100 U/mL penicillin and 100 μg/mL streptomycin; Gibco). All procedures were conducted under sterile conditions. Cultures were maintained in a humidified incubator at 37 °C with 5% CO_2_. Between days 7–10, cultures were shaken at 200 rpm for 2 h. The supernatant, containing detached microglia, was collected and plated into 96-, 24-, and 6-well plates at a density of 3 × 10^4^ cells/cm^2^. Mixed glial cultures were maintained for an additional 5 days, trypsinized, and seeded at the same density.

#### 4.1.2. Human iPSC-Derived Astrocyte Cultures

Human induced pluripotent stem cell (iPSC)-derived astrocytes were differentiated from neural stem cells (NSCs) obtained from four healthy donors at the D’Or Institute for Research and Education. Reprogramming was approved by the Ethics Committee of Hospital Copa D’Or, and experiments were conducted in compliance with regulations. NSCs were plated at a density of 5 × 10^4^ cells/cm^2^ in 75 cm^2^ flasks and differentiated as previously described. After 21 days, cells were exposed to astrocyte medium containing 10% FBS in DMEM/F12. At this stage, cells were referred to as iPSC-derived radial glia-like cells due to positive labelling for radial glial markers PAX6 and phosphorylated vimentin, which decrease over time in culture [34]. Cells were then expanded for an additional 4 weeks to enhance astroglial maturation and plated at 3 × 10^4^ cells/cm^2^ in 96-, 24-, and 6-well plates.

#### 4.1.3. Human Microglia C20 Cell Line

The C20 human microglial cell line was kindly provided by Dr. Henning Ulrich (Department of Biochemistry, Institute of Chemistry, University of São Paulo, USP). These cells were originally derived and characterized from fresh cortical tissue of adult patients undergoing brain surgery at university hospitals in Cleveland (Human Tissue Procurement Facility, UH) [35]. Cells were cultured in DMEM supplemented with 10% FBS and antibiotics (100 U/mL penicillin and 100 μg/mL streptomycin; Gibco) and plated at 3 × 10^4^ cells/cm^2^ in 96-, 24-, and 6-well plates after trypsinization.

#### 4.1.4. PC12 Neuronal Cell Culture and Differentiation

PC12 neuronal cells, derived from a murine adrenal pheochromocytoma was purchased from American Type Culture Collection (ATCC) (#CRL-1721.1 PC12 ADH, Rattus norvegicus, Manassas, VA, USA), serve as an in vitro neuronal model due to their capacity to extend neurites and exit the cell cycle upon stimulation with growth factors such as nerve growth factor (NGF) and fibroblast growth factor 1 (FGF1) [36,37]. PC12 cells were cultured in Petri dishes containing DMEM supplemented with 10% FBS, 10% EHS, 4 mM L-glutamine, and antibiotics. Cultures were maintained at 37 °C with 5% CO_2_. At confluence, cells were detached with 0.05% trypsin and 0.02% EDTA in PBS, centrifuged at 1000 rpm, and plated at 3 × 10^5^ cells/cm^2^ in 24- or 96-well plates. For differentiation, cells were treated with 100 ng/mL NGF (Sigma-Aldrich, Darmstadt, Germany) for 6 days, with the medium changed every 48 h.

### 4.2. Compounds and Treatments

To mimic KP-related neurotoxic conditions, cells were exposed to quinolinic acid (QUIN; 500 μM) or the IDO inhibitor 1-methyltryptophan (1-MT; 1.5 μM), both from Sigma-Aldrich. Fresh dilutions were prepared in serum-free culture medium. LPS (1 μg/mL) was used to induce glial activation, as previously described [12,16]. Agathisflavone (FAB) was extracted from the leaves of *C. pyramidale* Tul. as described previously [15,16,17], stored at 100 mM in DMSO, and diluted to 1 μM for treatments. Control cultures received equivalent DMSO (0.0001%). Glial cells (murine and human) were treated with QUIN (500 μM), 1-MT (1.5 μM), LPS (1 μg/mL), and/or FAB (1 μM), in the following combinations: FAB+LPS; FAB+QUIN; FAB+1-MT. After 24 h, conditioned media (CM) were collected for HPLC analysis or used to treat PC12 cells.

### 4.3. Rationale for Drug Concentrations

The concentrations of the compounds used in this study were selected based on previous literature and experimental optimization to ensure biological relevance and minimal cytotoxicity. Agathisflavone (FAB, 1 µM) was selected based on prior reports from our group demonstrating neuroprotective and anti-inflammatory effects in glial and neuronal cultures without compromising cell viability [15,16,17]. Lipopolysaccharide (LPS, 1 µg/mL) was used as a classical stimulus to induce glial activation, as previously described for astrocyte and microglial models [12,16]. Quinolinic acid (QUIN, 500 µM) was applied to mimic excitotoxicity mediated by N-methyl-D-aspartate (NMDA) receptor overactivation, a well-established concentration for modelling kynurenine pathway–related neurotoxicity [3,7,20]. The IDO1 inhibitor 1-methyl-D-tryptophan (1-MT, 1.5 µM) was used at a dose sufficient to achieve enzymatic inhibition while maintaining glial viability, as described in earlier studies [38]. All concentrations were validated in pilot assays to confirm reproducible induction of inflammatory and metabolic responses under the experimental conditions adopted in this work.

### 4.4. Analytical Methods

#### 4.4.1. MTT Cytotoxicity Assay

Cell viability was assessed using the the 3-(4,5-dimethylthiazol-2-yl)2,5-diphenyltetrazolium bromide (MTT) test (Thermo Fisher, Waltham, MA, USA). Cells were seeded in 96-well plates and, post-treatment, incubated with 100 μL MTT solution (0.5 mg/mL) in phenol red-free DMEM for 2 h at 37 °C. Then, 100 μL of lysis solution (20% SDS, 50% acetic acid, 2.5% 1M HCl) was added and incubated overnight at 37 °C. Formazan absorbance was measured at 540 nm using a Varioskan™ Flash plate reader (Thermo Fisher, Waltham, MA, USA). Results were expressed as mean ± SD of three independent experiments with eight replicates per condition.

#### 4.4.2. Quantification of L-Kynurenine as an Indirect Measure of IDO Activity

IDO activity was measured indirectly by quantifying the L-kynurenine levels in 160 μL of the culture medium (CM) from the different glial cell cultures after treatment, as described by Jesus et al. (2019) [38]. After adding 10 µL of 30% trichloroacetic acid per 160 µL of conditioned medium, the mixture was incubated at 50 °C for 30 min and centrifuged for 10 min at 600× *g* to precipitate kynurenine. One hundred microlitres (100 µL) of the supernatant was then transferred to 96-well plates, and 100 µL of 1.2% (*w*/*v*) 4-dimethylaminobenzaldehyde (Ehrlich’s reagent; Sigma Aldrich, Germany) diluted in glacial acetic acid was added and incubated for 10 min at room temperature. Absorbance was read at 492 nm using a Varioskan^TM^ Flash plate reader (Thermo Fisher, Waltham, MA, USA). Kynurenine concentrations were determined from standard curves (1–50 µg/mL) with L-ynurenine (K8625, Sigma Aldrich, Germany). Data were reported as % of control (or µg/mL).

#### 4.4.3. Immunofluorescence Analysis (GFAP, Iba1, Ki-67)

Cells were fixed with cold methanol (−20 °C, 10 min), permeabilized with 3% Triton X-100, and blocked with PBS + 1% BSA. Primary antibodies (anti-GFAP, anti-IBA1, anti-Ki-67) were applied overnight at 4 °C, followed by fluorophore-conjugated secondary antibodies (Alexa Fluor 488/594). Nuclei were stained with DAPI (5 μg/mL). Images were acquired using a Leica DFC7000 fluorescence microscope (Wetzlar, Germany). Negative controls omitted primary antibodies. The following primary and secondary antibodies were used at the indicated dilutions: rabbit anti-GFAP (1:200; ab68428, Cambridge, UK); rabbit anti-IBA1 (1: 200; Wako, 019-19741, Saitama, Japan), and mouse anti-Ki67 IgG (1:300, Abcam ab66155, Cambridge, UK); Alexa Fluor 488 conjugated goat anti-mouse IgG (1:500; Life Technologies, CA, USA), Alexa Fluor 594 conjugated goat anti-mouse IgG (1:500; Life Technologies, Carlsbad, CA, USA), Alexa Fluor 488 conjugated goat anti-rabbit IgG (1:500; Life Technologies, Carlsbad, CA, USA), Alexa Fluor 594 conjugated goat anti-mouse IgG (1:500, Life Technologies, Carlsbad, CA, USA).

#### 4.4.4. RT-qPCR for Gene Expression Analysis

Gene expression was assessed in murine primary astrocyte and microglia cultures by RT-qPCR. RNA was extracted using Trizol^®^ reagent (Invitrogen™, Life Technologies, Waltham, MA, USA), and cDNA was synthesized from 2.5 μg total RNA using VILO™ SuperScript^®^ Master Mix (Invitrogen™, Life Technologies, Waltham, MA, US). Reactions were performed using TaqMan^®^ Universal Master Mix II and probes (Invitrogen™, Life Technologies, Waltham, MA, USA) for Kmo (Rn01411937_m1), Kat2 (Rn00567882_m1), Arg1 (Rn00691090_ m1), Nos2 (Rn00561646_m1), Il-1b (Rn00580432_m1), and Actb (Rn00667869_m1) (endogenous control). RT-qPCR was run on QuantStudio™ 7 Flex under standard conditions. Data were analyzed using the 2^−ΔΔCt^ method and GraphPad Prism v8.4.

#### 4.4.5. HPLC Analysis of Kynurenine Pathway Metabolites

Metabolites were analyzed by HPLC (Agilent G7161A with DAD at 280 nm, Agilent, Santa Clara, CA, USA). Separation used a gradient with mobile phase A (water + 0.1% formic acid) and B (methanol + 0.1% formic acid) at 0.8 mL/min:

- 0–20 min: 3–25% B
- 20–22 min: 100% B
- 22–25 min: 100% B
- 25–29 min: 3% B

Standards (1 mg/mL) included TRP, 1-MT, KYNA, and QUIN. Samples and standards were filtered (0.45 μm) and run in triplicate. Compounds were identified by retention time and UV-Vis spectra.

### 4.5. Statistical Analysis

Data were analyzed using GraphPad Prism v10. Results are presented as mean ± SEM from at least three independent experiments. One-way ANOVA with Student–Newman–Keuls post-test was used for parametric data and the Bonferroni test for non-parametric data. Significance was set at *p* < 0.05 (95% confidence interval).

## 5. Conclusions

This study shows that FAB, a flavonoid isolated from *P. pyramidalis*, acts as an effective modulator of tryptophan metabolism, glial reactivity, and glia–neuron communication. Using integrated functional, molecular, and chromatographic analyses, we found that FAB beneficially modulates pathways linked to chronic inflammation and neurotoxicity, supporting its pharmacological potential for the prevention or treatment of neurodegenerative disorders.

FAB attenuated the reactive profile of astrocytes challenged with LPS or QUIN, two well-established glial activators associated with neuroinflammation. In neuronal culture models, conditioned media from FAB-treated astrocytes preserved the viability and morphology of differentiated PC12 cells under inflammatory challenge, underscoring FAB’s neuroprotective properties. These findings suggest that FAB functions not only as an anti-inflammatory agent but also as a modulator of paracrine signalling within the neuroglial network.

Our data further indicate that FAB interferes with the KP by modulating key enzymes, including IDO and KMO. Inhibition of these enzymes, particularly when FAB was combined with the IDO inhibitor 1-MT, was associated with enhanced glial viability and preserved morphology [18], consistent with a direct interaction between FAB and components of Trp metabolism catabolism or downstream metabolite-mediated signalling. FAB’s effects were stimulus-dependent and dynamically modulated. When combined with either LPS or QUIN, FAB produced differential regulation of inflammatory and astrocytic responses, indicating a context-sensitive and adaptable mode of action. This functional plasticity could be leveraged to design targeted therapies tailored to the neuroinflammatory microenvironment [44].

Taken together, these results position FAB as a promising candidate for modulating neuroinflammation and neurotoxicity via the KP, with emphasis on preserving glial function and neuronal integrity. The combination of FAB with IDO inhibitors represents a natural-compound-based strategy worthy of further exploration. Future studies should validate these effects in vivo and explore bioactive formulations to enhance FAB’s bioavailability and therapeutic reach.

## Data Availability

The original contributions presented in this study are included in the article/Supplementary Materials. Further inquiries can be directed to the corresponding author(s).

## Supplementary Materials

The HPLC chromatographic profile of KP metabolites supporting information can be downloaded at: https://www.mdpi.com/article/10.3390/ijms262411951/s1.

## Abbreviations

The following abbreviations are used in this manuscript:

FAB	Agathisflavone
IDO	Indoleamine 2,3-Dioxygenase
KMO	Kynurenine Monooxygenase
KAT	Kynurenine Aminotransferase
KP	Kynurenine Pathway
QUIN	Quinolinic Acid
KYNA	Kynurenic Acid
LPS	Lipopolysaccharide
1-MT	1-Methyl-D-Tryptophan
hAst	Human iPSC-derived Astrocytes
GFAP	Glial Fibrillary Acidic Protein
IL-10	Interleukin-10
iNOS	Inducible Nitric Oxide Synthase
ARG 1/2	Arginase ½
MTT	Methylthiazolyldiphenyl-tetrazolium bromide assay
HPLC	High Performance Liquid Chromatography
CM	Conditioned Medium
MCM/ACM	Microglia-/Astrocyte-conditioned Medium
