# Supplementary figures and images for: Agathisflavone Modulates the Kynurenine Pathway and Glial Inflammatory Responses with Implications for Neuroprotection
Source: Int J Mol Sci. 2025 Dec 11;26(24):11951. doi: 10.3390/ijms262411951 (PMC12733300; doi:10.3390/ijms262411951)

Chromatograms and spectra with analytical standard

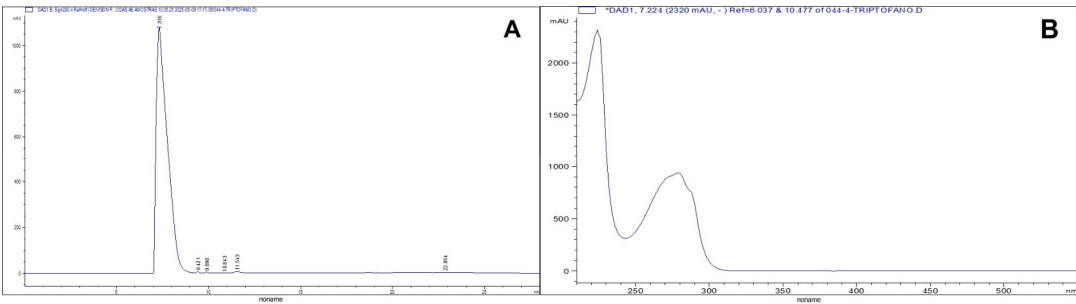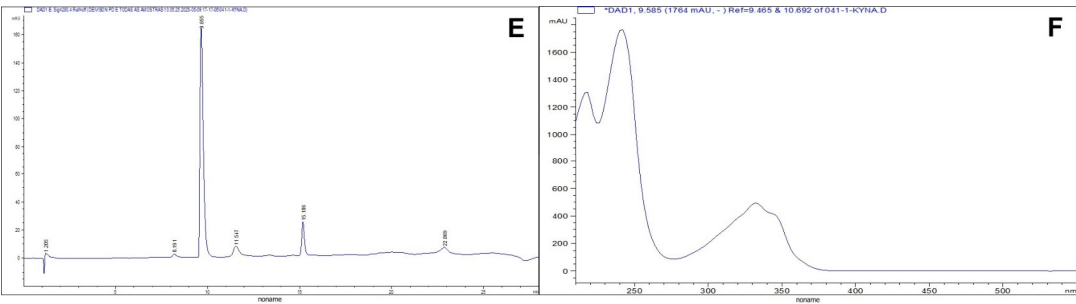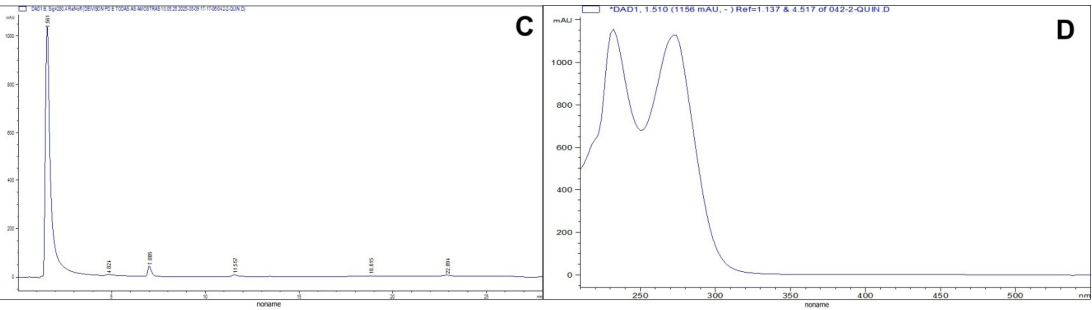

Supplement: Supplementary file 1 [file ijms-26-11951-s001.zip › ijms-3927366-supplementary.pdf]
